# FIP1L1–PDGFRα-Positive Loeffler Endocarditis—A Distinct Cause of Heart Failure in a Young Male: The Role of Multimodal Diagnostic Tools

**DOI:** 10.3390/diagnostics13101795

**Published:** 2023-05-19

**Authors:** Andreea Varga, Diana Andreea Moldovan, Marian Pop, Istvan Benedek, Attila Kövecsi, Robert Adrian Dumbrava, Dragos Gabriel Iancu, Liviu Cristescu, Laurentiu Huma, Ioan Tilea

**Affiliations:** 1 Department ME2-Clinical Disciplines, George Emil Palade University of Medicine, Pharmacy, Science and Technology of Targu Mures, 540142 Targu Mures, Romania; andreea.varga@umfst.ro; 2 Department of Internal Medicine II-Cardiology, Emergency Clinical County Hospital, 540042 Targu Mures, Romania; 3 Department of Cardiology I, The Emergency Institute for Cardiovascular Diseases and Transplantation, 540136 Targu Mures, Romania; diana.moldovan@umfst.ro (D.A.M.); laurentiu.huma@umfst.ro (L.H.); 4 Department ME1-Preclinical Disciplines, George Emil Palade University of Medicine, Pharmacy, Science and Technology of Targu Mures, 540142 Targu Mures, Romania; marian.pop@umfst.ro; 5 Department of Radiology and Medical Imaging, The Emergency Institute for Cardiovascular Diseases and Transplantation, 540136 Targu Mures, Romania; 6 Department of Family Medicine, George Emil Palade University of Medicine, Pharmacy, Science and Technology of Targu Mures, 540142 Targu Mures, Romania; benedek.istvan@umfst.ro; 7 Department of Hematology II, Emergency Clinical County Hospital, 540042 Targu Mures, Romania; 8 Department of Pathology, George Emil Palade University of Medicine, Pharmacy, Science and Technology of Targu Mures, 540142 Targu Mures, Romania; attila.kovecsi@umfst.ro; 9 Department of Pathology, Emergency Clinical County Hospital, 540136 Targu Mures, Romania; 10 Doctoral School, George Emil Palade University of Medicine, Pharmacy, Science and Technology of Targu Mures, 540142 Targu Mures, Romania; robert-adrian.dumbrava@umfst.ro (R.A.D.); dragos-gabriel.iancu@umfst.ro (D.G.I.); liviu.cristescu@umfst.ro (L.C.); 11 Department of Internal Medicine VIII, George Emil Palade University of Medicine, Pharmacy, Science and Technology of Targu Mures, 540142 Targu Mures, Romania; 12 Department of Cellular and Molecular Biology, George Emil Palade University of Medicine, Pharmacy, Science and Technology of Targu Mures, 540142 Targu Mures, Romania

**Keywords:** heart failure, hypereosinophilic syndrome, Loeffler endocarditis, FIP1L1–PDGFRA fusion gene, cardiac imaging

## Abstract

The presence of the Fip1-Like1-platelet-derived growth factor receptor alpha (FIP1L1–PDGFRα) fusion gene represents a rare cause of hypereosinophilic syndrome (HES), which is associated with organ damage. The aim of this paper is to emphasize the pivotal role of multimodal diagnostic tools in the accurate diagnosis and management of heart failure (HF) associated with HES. We present the case of a young male patient who was admitted with clinical features of congestive HF and laboratory findings of hypereosinophilia (HE). After hematological evaluation, genetic tests, and ruling out reactive causes of HE, a diagnosis of positive FIP1L1–PDGFRα myeloid leukemia was established. Multimodal cardiac imaging identified biventricular thrombi and cardiac impairment, thereby raising suspicion of Loeffler endocarditis (LE) as the cause of HF; this was later confirmed by a pathological examination. Despite hematological improvement under corticosteroid and imatinib therapy, anticoagulant, and patient-oriented HF treatment, there was further clinical progression and subsequent multiple complications (including embolization), which led to patient death. HF is a severe complication that diminishes the demonstrated effectiveness of imatinib in the advanced phases of Loeffler endocarditis. Therefore, the need for an accurate identification of heart failure etiology in the absence of endomyocardial biopsy is particularly important for ensuring effective treatment.

## 1. Introduction

Hypereosinophilic syndrome (HES) represents a complex diagnosis involving a broad field of disorders in which the main role of the pathological pathway is attributed to eosinophilic cells, which are associated with hematological malignancies and reactive causes [1]. The diagnosis of HES includes the confirmation of organ damage in addition to the primary criteria of a peripheral eosinophilic blood count of >1500 cells/m^2^, as is accepted by the current guidelines and consensus [1,2].

Organ damage in the presence of hypereosinophilic states is based on multimodal action of the molecules that are released by activated eosinophilic cells [3]. Persistent eosinophilia in the blood flow leads to infiltrative lesions affecting various territories (heart, lungs, liver, skin, etc.), which result in different degrees of dysfunction or secondary complications [3,4,5,6]. Cardiac involvement, known as Loeffler disease, alters the structure of the endothelium and is associated with a high probability of intracardiac thrombus formation.

In 2020, a critical review of Mattis et al. provided a classification of hypereosinophilia, including four main categories in respect to the prevailing etiologies: primary (neoplastic HE), secondary (nonneoplastic conditions and paraneoplastic disorders), familial, and idiopathic conditions. The main causes of neoplastic hypereosinophilia are associated with chronic eosinophilic leukemia not otherwise specified, myeloid/lymphoid neoplasms with eosinophilia and gene rearrangement (PDGFRA, PDGFRB, FGFR1, JAK2, FLT3, ABL1), myeloproliferative neoplasm with eosinophilia (e.g., CML and JAK2 V617F+ MPN), acute myeloid leukemia with inv(16) or t(16;16)/CBFB-MYH11, myelodysplastic syndrome with eosinophilia, myelodysplastic syndrome/myeloproliferative neoplasm with eosinophilia, and aggressive systemic mastocytosis with eosinophilia [7].

In the revised 2022 World Health Organization (WHO) classification of eosinophilic disorders, diagnostic criteria were established relating to Fip1-Like1-platelet-derived growth factor receptor alpha (FIP1L1–PDGFRα) gene rearrangement when it is associated with hypereosinophilia (HE). Specifically, the criteria require the presence of a myeloid neoplasm with predominant eosinophilia and evidence of a FIP1L1–PDGFRα fusion gene, which can be detected by fluorescence in situ hybridization (FISH) or reverse transcription polymerase chain reaction [2]. Studies regarding this disorder reveal the prevalent distribution of this syndrome in male patients with no apparent molecular or physiopathological basis [5,8,9]. The presence of FIP1L1–PDGFRα fusion in individuals with HES helps in predicting a possible response to imatinib (a tyrosine kinase inhibitor) treatment, which is capable of suppressing the fusion gene [8,9,10].

The aim of this paper is to emphasize the importance of accurate diagnosis and the use of multimodal diagnostic tools in describing the cardiac involvement of HES when it is secondary to chronic myeloid leukemia with positive FIP1L1–PDGFRα fusion—specifically in the context of a young male patient who presented the symptoms and signs of heart failure.

## 2. Case Presentation

A 26-year-old Caucasian male patient, who was also a smoker, was admitted for exertional dyspnea, fatigue, and bilateral lower limb edema, which had progressively worsened one week prior to admission. Besides having a history of tobacco use, the patient denied any medical history or medication. Furthermore, he described his symptoms as beginning two months before presentation, with continual progression. A pneumological exam (clinical evaluation, pulmonary functional tests, and thoracic computed tomography (CT) was performed in ambulatory settings and raised the suspicion of asthma, and diffuse pulmonary fibrosis was diagnosed. At admission, the clinical examination indicated a mildly elevated blood pressure (135/100 mmHg), tachycardia (heart rate of 93 beats per minute, i.e., bpm), bilateral edema of the lower limbs, and hepatomegaly.

**Figure 1:**
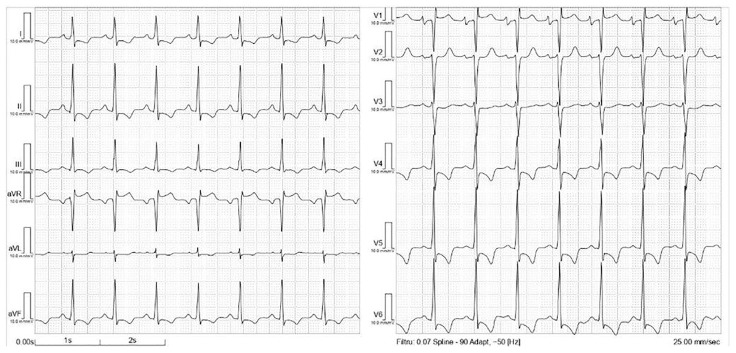
Resting electrocardiogram—sinus rhythm, normal QRS axis, incomplete RBBB, and negative T waves in lateral and inferior leads.

Initial laboratory data showed a total white blood count with significant eosinophilia (i.e., 42.7% of the white blood cells), mild anemia (hemoglobin of 11.9 g/dL, hematocrit 36%), mild thrombocytopenia (128.000/mm^3^), and normal electrolyte levels as well as renal and liver function. Acute inflammatory reactants (erythrocyte sedimentation rate and fibrinogen) were within normal limits. The baseline N-terminal pro-B-type natriuretic peptide (NT-proBNP) value was 6541 pg/mL (cut-off value <125 pg/mL), and a lactate dehydrogenase value of 1000 U/L was also detected. The electrocardiogram illustrated sinus tachycardia with 93 bpm and a normal QRS axis; however, an incomplete right bundle branch block, as well as negative T waves in lateral and inferior leads, were also detected (see Figure 1).

**Figure 2:**
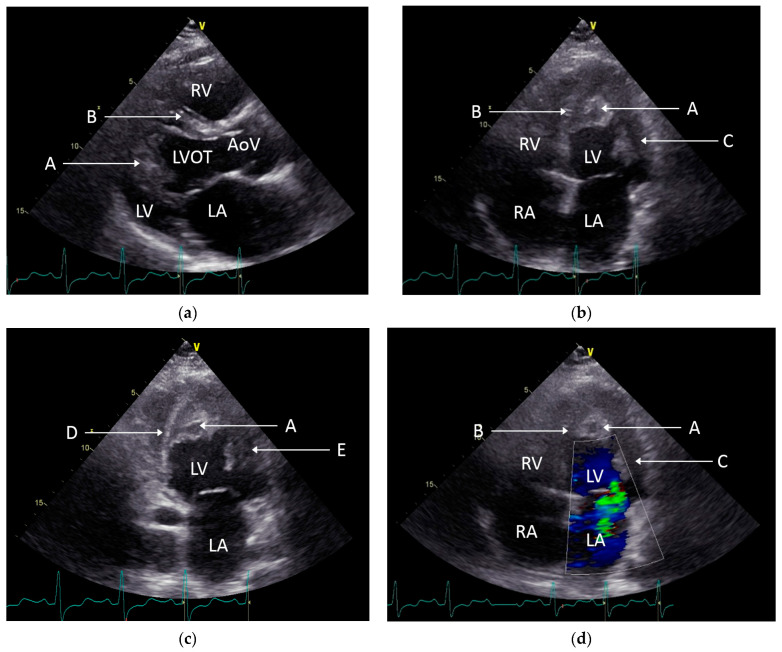
Bidimensional TTE images. (**a**) The parasternal long axis revealing the presence and extension of the intraventricular mass towards the posterior wall and subvalvular apparatus. (**b**) Apical four-chamber view (A4C) depicting the hyperechogenic structure obliterating the apex and lateral wall. (**c**) Apical two-chamber view (A2C) showing the extent of the intraventricular mass to the anterior wall of LV. (**d**) A4C and color doppler showing moderate-to-severe mitral regurgitation. A—intraventricular mass, AoV—aortic valve, B—interventricular septum, C—lateral wall of the left ventricle, D—inferior wall of the left ventricle, E—anterior wall of the left ventricle, LA—left atrium, LV—left ventricle, LVOT—left ventricular outflow tract, RA—right atrium, and RV—right ventricle.

Two-dimensional transthoracic echocardiography (TTE) disclosed a mildly reduced left ventricular ejection fraction (LVEF:45%, Simpson formula), moderate-to-severe mitral regurgitation, minimal pericardial effusion, and hyperechogenic masses that encompassed approximately 50% of both the ventricular cavities (see Figure 2). The TTE findings raised the question of Loeffler endocarditis in the presence of hypereosinophilia.

The coronary CT angiogram revealed a normal coronary anatomy with no atherosclerotic lesions or anatomic anomalies, but it did raise a suspicion of Loeffler endocarditis in the presence of biventricular thrombi; in addition, the contrast-enhanced thoraco-abdominal CT scans revealed small mediastinal adenopathic masses, multiple splenic infarctions (maximum size of 37/10 mm), and minimal ascites, results that were in accordance with those of the abdominal ultrasound.

**Figure 3:**
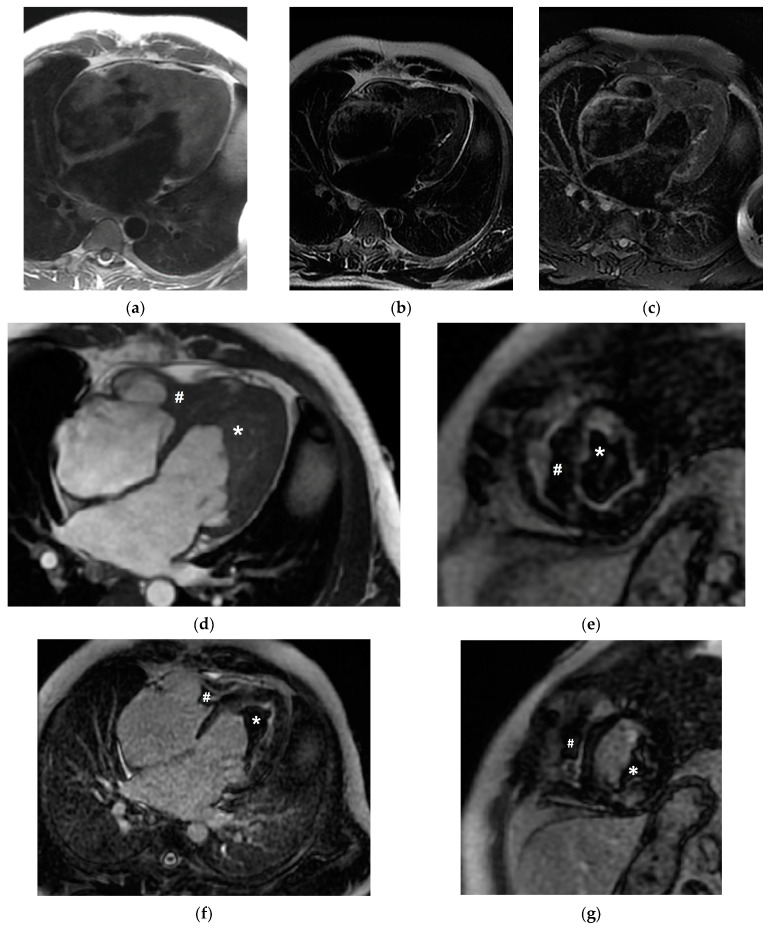
CMR images revealing left (*) and right (#) intraventricular masses. (**a**) T1-weighted 4C view. (**b**) T2-weighted 4C view. (**c**) T2-weighted fat-suppressed 4C view. (**d**) 4C-balanced free steady-state precession 4C view. (**e**,**g**) Late gadolinium-enhanced SA view. (**f**) Late gadolinium-enhanced 4C view.

To better characterize the ventricular mass, cardiac magnetic resonance imaging (CMR) was scheduled. An amorphous mass was identified, whereby it occupied the left ventricular apex and was found to extend into the inferior and inferolateral medio-cardiac segments, with overall dimensions of 7 cm × 5 cm × 4.7 cm. The apex of the right ventricle was found to be filled by a similar mass, which extended and covered the papillary muscles but had smaller dimensions. Neither the mass nor the adjacent myocardium showed signal changes suggestive of edema; further, since the native characterization of the mass was nonspecific, late gadolinium enhancement (LGE), which was conducted following contrast administration, demonstrated the lack of enhancement of the masses, with strong enhancement of the endocardial contours adjacent to the masses, thereby indicating fibrosis (see Figure 3). The CMR characteristics of the ventricular masses suggested a thrombotic structure, and an anticoagulation enoxaparin-weight-adjusted dose regimen was initiated alongside high doses of corticosteroids in order to suppress the hematological disorder.

The presence of parasitic infections, allergies, or adrenal deficiency was excluded following additional assessments. The presence of an autoimmune disease as the secondary cause of eosinophilia was also ruled out (negative ANCA, ANA, normal values for the complement complex, and a negative Coombs test).

The hematological findings from a peripheral blood smear were 16% eosinophilic cells, 57% neutrophils, 18% lymphocytes, 42% eosinophilic cells, 30% neutrophils, 24% lymphocytes, 0% basophilic cells, and bone marrow biopsy-revealed anisocytosis. According to the recommendations, analyses were conducted to determine the status of JAK-2 mutation, bcr-abl, and FIP1L1–PDGFRα fusion, of which a positive result for the presence of the FIP1L1–PDGFRα fusion gene was returned via FISH.

Hereafter, a diagnosis of HES—defined as myeloid neoplasm with FIP1L1–PDGFRα rearrangement with an aspect of Loeffler endocarditis complicated with heart failure (HF)—was established two months after the patient’s first presentation. As such, targeted therapy with a tyrosine kinase inhibitor (TKI-imatinib 400 mg/day) was initiated alongside preexistent HF regimen, which was administered since the first week of index admission, thereby replacing pre-treatment with corticosteroids. Initially, the TKI therapy was well tolerated by the patient; however, one month later, there was a relapse in HF symptomatology and the patient experienced intolerable digestive symptoms (diffuse abdominal pain, nausea, and vomiting). Echocardiography revealed a slight reduction in the extent of intraventricular mass. Still, following hematologist advice, the imatinib dose was reduced, and TKI administration eventually halted due to undesirable gastrointestinal side effects. Despite extensive treatment (including parenteral anticoagulation) and careful follow-up, the cardiac impairment progressively worsened, and there were additional complications of sepsis, multiple embolization (spleen, kidney, brain, and acute occlusion of terminal aorta), as well as a severe thrombocytopenia, which led to patient death.

**Figure 4:**
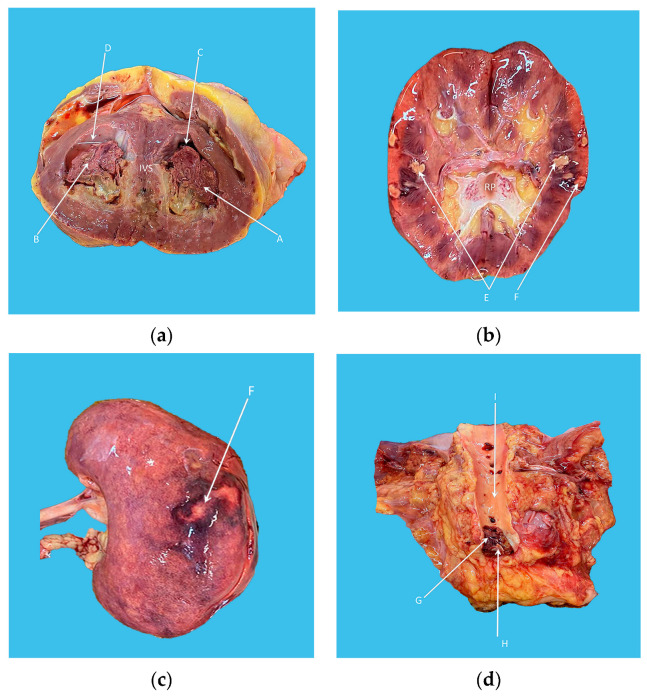
(**a**) Organized intraventricular thrombi (A and B) are seen in the left (C) and right ventricle (D), respectively. (**b**,**c**) Medullar (E) and cortical (F) infarcted areas of the left kidney. (**d**) Infrarenal aorta (I) with a large saddle thrombus (G) on the aortic bifurcation (H). IVS—interventricular septum and RP—renal pelvis.

The pathology examination revealed biventricular hypertrophy associated with endomyocardial fibrosis and extended thrombi, as well as diffuse infarcted areas of the spleen, brain, and left kidney; this was in addition to the presence of a large infrarenal aortic saddle thrombus (see Figure 4). A microscopic examination of the bone marrow indicated preserved histological structure with a subtle increase in the number of eosinophilic cells.

## 3. Discussion

Myeloid neoplasm with FIP1L1–PDGFRα gene rearrangement is an uncommon cause of HE consisting of peripheral eosinophilia, defined as an elevation of the eosinophil count above 1.5 × 10^3^/uL, whereby the presence of the FIP1L1–PDGFRα fusion gene is identified via the FISH technique [10]. The genotype of these patients includes a 4q12 deletion that leads to fusion between the FIP1L1 and PDGFRα genes, which in turn results in the expression of an active tyrosine kinase that is involved in the proliferation of eosinophilic cells [11]. Despite limited available data, the fusion gene is reported to be found in 10% to 60% of hypereosinophilia cases [8,12]. Moreover, hypereosinophilic syndrome is associated with myeloid proliferation and is predominantly identified in male patients in up to 80–90% of the studied cohorts [5,8,9,13]. Still, molecular or pathological arguments supporting this evident deviation towards male patients has not yet been mentioned or explained.

A comprehensive clinical profile is difficult to define, since the number of reported cases is limited, and the symptoms are usually not disease specific. However, an important aspect that needs to be emphasized is that the heterogeneity of symptoms is dependent on the involved organ. Once the diagnosis of HES is established, it is essential to differentiate the main etiologies in order to offer patient-targeted therapy in a timely fashion [14]. After HES is determined based on the complete blood count or in the presence of high clinical suspicion, peripheral blood and bone marrow smears are mandatory components of the diagnostic algorithm [10,15]. Tailored imagistic modalities (i.e., ultrasound exams, computed tomography, or magnetic resonance imaging) are useful for identifying organ involvement and other features (secondary sites, complications, etc.). Furthermore, they provide significant details even in the early phases of the structural alteration and may be able to support a diagnosis in the absence of morphopathological confirmation.

Organ involvement was noticed in 19% to 91% of cases of established hypereosinophilia, depending on the number of patients included in studies [13,16]. In these patients, tissue damage of the heart, as one of the prevailing organs, has a reported incidence of 34% to 75%, which leads to significant mortalities due to the extent of lesions and the development of consequent, progressive heart failure [8,17].

The first description of cardiac involvement in a patient with hypereosinophilia was provided by Wilhelm Loeffler in 1936. Loeffler endocarditis (LE) is considered a rare but aggressive complication of hypereosinophilia, described by most authors as consisting of three stages. First, there is eosinophilic infiltration of the endocardial tissue, where the eosinophils release mediators and cytotoxic molecules with subsequent local arteriolar necrosis, followed by thrombus formation on the exposed affected endothelium. The third stage involves fibrotic remodeling that generates restrictive cardiomyopathy [3,18]. In our case, the laboratory findings suggest reflected systemic inflammatory states and cardiac damage. By complementing the results of clinical presentation and cardiac imaging studies, NT-pro BNP is considered essential for an exhaustive diagnosis of heart failure.

Transthoracic and transesophageal echocardiography represent appropriate noninvasive tools for the purposes of evaluating cardiac structure and function. In addition, they also uncover the alterations that are induced by the hypereosinophilic state [19]. Thrombus formation may affect the subvalvular apparatus with secondary regurgitation or transvalvular occlusions by the large emboli. Endomyocardial infiltration and fibrosis lead to impaired diastolic function, which is the main reason for the clinical features of heart failure. According to the published reports, these individuals often have a restrictive echocardiographic pattern, most likely following the fibrotic phase. Polito et al., in their state-of-the-art review, acknowledged that endomyocardial thickening of the cardiac apex and/or the presence of ventricular thrombi were the most common echocardiographic findings that suggest LE, specifically in the context of clinical suspicion of HES [20]. In our case, TTE had a pivotal role in identifying the intraventricular masses that raised suspicion of thrombi, which was later supported by the CMR evaluation. The extension of the mass appeared to involve the papillary muscles and triggered the development of mitral regurgitation.

Cardiac magnetic resonance imaging is the mainstay noninvasive modality for obtaining an accurate description of endomyocardial structure. In addition, it provides the possibility of distinguishing between myocardial edema and fibrosis [21,22,23]. In the absence of endomyocardial biopsy (EMB), echocardiography and CMR play a pivotal role in the diagnostic workup of Loeffler endocarditis, leading to the initiation of suitable treatment [24]. In patients with confirmed HES, computed tomography is used to assess multiorgan involvement or the recognition of embolization originating from cardiac thrombi.

Although noninvasive cardiovascular imaging offers valuable information, EMB represents the gold standard in the diagnostic algorithm, despite its invasiveness and the possibility of it incurring multiple potential complications [25]. The main suggestive histologic alteration is represented by predominant interstitial infiltration into eosinophilic cells, whereby edema may be exposed in the acute phase. In our case, EMB could not be performed due to the substantial risk of embolization as a consequence of the localization and expansion of the mass.

Our paper emphasizes the necessity of multimodal imaging in order to acquire a more precise and refined diagnosis, as well as the necessity of determining a comprehensive description of multiorgan involvement. The patient-directed selection of imaging techniques, alongside laboratory tests, critically contributes to the etiological diagnosis of heart failure and, thereby, establishing an appropriate drug regimen.

Differential diagnoses between HES etiologies may be challenging, and the complexity of this entity can lead to delays in the initiation of treatment. The treatment of hypereosinophilic syndrome implies a correct diagnosis, but glucocorticoids are widely used as a first option [17]. One study, including 188 subjects who were diagnosed with HES of multiple etiologies, revealed that 85% of the patients who received corticosteroid monotherapy exhibited complete or partial responses; however, this is still considered a better option for the FIP1L1–PDGFRα-negative group of patients [26]. Isolated cases of HES with the FIP1L1–PDGFRα fusion gene have been described in the literature as positively responding to prednisone [27]. As in our case, steroids (initially prednisolone intravenous, followed by prednisone) were the initial therapeutic option until molecular testing was performed to ascertain the presence of FIP1L1–PDGFRα.

Imatinib mesylate, a tyrosine kinase inhibitor, is shown to be an effective treatment option for carriers of the FIP1L1–PDGFRα gene [10]. Small studies have suggested complete hematological or molecular remissions under the administration of imatinib after months or years of treatment [8,9,28]. Furthermore, the current data indicate that for certain patients, there is a dose-related long-term response, i.e., 400 mg rather than 100–200 mg per day of imatinib resulted in a better outcome [29]. Previous reported cases of myeloid neoplasm connected to the hybrid gene highlighted a complete hematological and molecular response, even in the presence of peripheral blast cells [30].

Without accurate diagnosis and targeted treatment, the prognostic of this rare disorder is poor, leading to death caused by systemic complications involving multiple organ damage. However, early detection and exhaustive evaluation in order to ascertain multiple organ involvement is crucial for the patient in the era of TKI treatment [31]. Relapsing after a period of reduced dose or after stopping the treatment highlights the idea that imatinib has a suppressive effect over the gene, rather than determining its abolition [6]. Following TKI treatment, a 5-year survival rate of 93.5% was reported in a single Chinese center [9]. Moreover, 2-year survival was noticed in a reduced cohort after ceasing TKI therapy, following complete molecular remission [8].

Data regarding the response to imatinib in patients with organ involvement are insufficient; however, certain case series have suggested a positive effect on partial or complete remission after weeks of treatment [9,32,33]. Cardiac alteration and the development of eosinophilic endocarditis may be identified in later phases when a mortality of 35% to 50% is reported [23]. Limited cases with cardiac involvement were reported to undergo remission based on the image findings [34]. Furthermore, the early detection of cardiac involvement, i.e., while the structural alterations are still reversible, is essential for initiating hematological treatment and may have positive prognostic value [35]. Intracardiac thrombus regression was reported in 42.4% of the cases included in one literature review related to Loeffler endocarditis, and the idea of an additional anticoagulation regimen was considered of foremost importance in the outcome and evolution of these patients [36].

Helbeg et al. published the results of a 12-year follow-up on patients who expressed FIP1L1–PDGFRα rearrangement and reported positive results subsequent to a treatment of imatinib. However, the study also cited one case of death due to heart failure, despite experiencing complete hematological and molecular responses [28]. Considering that there was an improvement in the hematological status of our patient after the initiation of imatinib, confirmed afterwards by the microscopic examination of the bone marrow during necropsy, there may a similarity to the case described by Helbig et al. [28]. The presence of heart failure and systemic complications led to progressive worsening in the clinical state of our patient. To the best of our knowledge, the scientific literature concerning HES is scarce, this case being one of the few documented cases of HES with positive FIP1L1–PDGFRα fusion and severe cardiac impairment. The absence of extensive research on this topic, as well as the reduced number of diagnosed and reported patients with HES associating heart complications, constitutes a barrier to comparing different treatment approaches.

Molecular studies on imatinib effects raise awareness on the possibility of inducing other hematological disorders by inhibiting hematopoiesis [12]. Unfortunately, our case draws attention to the complications that are caused by the frailty of these patients, independent of eosinophil serum levels, which were normalized under suppressive therapy. A multidisciplinary approach and follow-up are essential for the purposes of complex evaluation and informing management decisions.

## 4. Conclusions

Hypereosinophilic syndrome represents a complex condition for which a multidisciplinary approach is required to establish the etiology, and this should be followed up with the most appropriate treatment as soon as possible. The FIP1L1–PDGFRα gene rearrangement that is associated with myeloid neoplasm is one of the foremost causes of HES, and it has been noted that there is a positive response to imatinib as the first-line treatment for HES. Nevertheless, the development of heart failure and Loeffler endocarditis with peripheral embolization secondary to cardiac involvement represents critical complications that diminish the demonstrated effectiveness of imatinib in the context of adult hypereosinophilic syndrome. This case report highlights the potential fatal cardiac complications independently of the progression of FIP1L1–PDGFRα myeloid leukemia.

## Data Availability

Additional information can be obtained from the corresponding author upon reasonable request.
